# Online technology use in physiotherapy teaching and learning: a systematic review of effectiveness and users’ perceptions

**DOI:** 10.1186/s12909-015-0429-8

**Published:** 2015-09-28

**Authors:** Aleksandra K. Mącznik, Daniel Cury Ribeiro, G. David Baxter

**Affiliations:** Centre for Health, Activity, and Rehabilitation Research, School of Physiotherapy, University of Otago, Otago, New Zealand

## Abstract

**Background:**

The use of online technologies in health professionals’ education, including physiotherapy, has been advocated as effective and well-accepted tools for enhancing student learning. The aim of this study was to critically review the effectiveness, and user perceptions of online technology for physiotherapy teaching and learning.

**Methods:**

Following databases were systematically searched on the 31^st^ of August 2013 for articles describing implementation of online technologies into physiotherapy teaching and learning: ERIC, CINAHL, Web of Science, Academic search complete, ProQuest Nursing and Allied Health Source, Medline, Embase, and Scopus. No language, design or publishing date restrictions were imposed. Risk of bias was assessed using the 2011 Mixed Methods Appraisal Tool checklist (MMAT).

**Results:**

A total of 4133 articles were retrieved; 22 articles met the inclusion criteria and were accepted for final analysis: 15 on the effectiveness of technology, and 14 on users’ perceptions. Included studies used three designs: case study (14 articles), controlled trial (3), and randomized controlled trial (5). Studies investigated both pre-registration physiotherapy students (1523) and physiotherapy professionals (171). The quality of studies ranged from 67 to 100 % on the MMAT checklist which can be considered moderate to excellent. More than half of the studies (68 %) received scores greater than 80 %. Studies typically investigated websites and discussion boards. The websites are effective in enhancing practical skills performance, and discussion boards in knowledge acquisition, as well as in development of critical and reflective thinking. Students’ perceptions of the use of websites were mostly positive, providing students with entertaining, easy accessible resources. Perceived barriers to the use of websites included difficulties with internet connection, insufficiently interactive material, or personal preference for paper-based materials. Discussion boards were perceived as deepening students’ thinking and facilitating reflection, allowing for learning from multiple perspectives, and providing easy communication and support.

**Conclusions:**

The results of this review suggest that online technologies (i.e., websites and discussion boards) have many benefits to offer for physiotherapy teaching and learning;

There was minimal evidence of barriers for the use of online technologies, however, addressing the identified ones could enhance adherence to use of online technologies in health professionals’ education.

## Background

Online technologies can be defined as any service and communication tools available on or utilising the internet including social networks, web-based resources and discussion boards. Online technologies have become an indispensable part of students and academics’ life in higher education, influencing strategies for learning [1–3]. Students in the health professions seem to access the internet daily, as part of which they are engaged in diverse online activities, focused mainly around social media platforms [4]. The rapid evolution of web-based information platforms, and social media in particular, has made the internet the primary source of information for many health professions students [5], meaning that medical textbooks and paper-based materials are no longer the main source of knowledge for student learning. Such embracing of online technologies into health professionals’ education is considered as inevitable and desired by some authors [2, 6], but more recently others advise caution and moderation [7, 8]. It is therefore important and timely to review and critically appraise the evidence from the empirical studies.

The broader incorporation of online technology into health professionals’ education is increasingly advocated, with claimed benefits including incorporation of quality content, support of life-long learning, flexibility of access, enrichment and personalisation of learner experience, and improved communication networks [9–11]. However, the use of online technologies as teaching tools has shown mixed results (benefits or no difference to traditional methods in facilitating knowledge or skills acquisition) in health professionals’ education. Previous reviews have focused on the use of technology in dental [11], medical [12], nursing education [13], and also for health professions’ faculty development [14]. To date however, there is a lack of reviews focusing on the use of online technology in physiotherapy education, and reviews conducted on mixed health professionals groups frequently do not include physiotherapy studies [15, 16]. Therefore a systematic review of the outcomes of online technology use in physiotherapy teaching was indicated to inform conclusions about their usefulness.

The aim of this study was twofold. First, we aimed to assess current evidence for the effectiveness of technology in physiotherapy teaching and learning in enhancing students’ skills and knowledge. Second, we aimed to summarize perceptions of physiotherapy students (or professionals) on the use of online technology.

The research questions were:

1. What is the effectiveness of technology on teaching and learning in physiotherapy in respect to student learning outcomes (i.e., students’ grades)?
2. What are the users’ perceptions (physiotherapy students and professionals) of benefits and barriers for use of technology in physiotherapy teaching and learning?

## Method

PRISMA guidelines were used to design and report this systematic review [17].

### Eligibility criteria

Articles published in peer-reviewed journals describing implementation of any online technology for teaching and learning in physiotherapy (Am: physical therapy) were included in this review. Articles could present designs such as case study (CS), controlled trial (CT), or randomised controlled trial (RCT). No restrictions on publication date or language were imposed.

Articles were excluded if: in studies with wide professional focus, results of the physiotherapy sample were impossible to separate from those for other allied health professions; at least one of effectiveness of, or perceptions of, online technologies were not investigated in the study; or if articles were of a review type.

For the purpose of this review, we adopted a pragmatic approach, and used effectiveness in a broad sense. Studies could investigate the effect of any online technology on knowledge or skills acquisition, engagement and participation, critical thinking and similar. Perceptions of online technologies were defined as mainly qualitative comments provided by physiotherapy students and practitioners in the studies.

### Search strategy

To capture maximum data and reduce bias in this review, a systematic search methodology was used to identify relevant articles. The systematic searches were performed on the 31^st^ of August 2013 in the following databases: ERIC, CINAHL, Web of Science, Academic search complete, ProQuest Nursing and Allied Health Source, Medline, Embase, and Scopus. A combination of key words related to the ‘technology’ and ‘physiotherapy’ was used. An example of the search strategy is presented in Table 1.

**Table 1:** Search strategy for Medline database

(new media OR social media OR social network* OR social sit* OR online network* OR online communit* OR online discussion* OR online participation* OR “web 2.0” OR mobile technolog* OR handheld device* OR digital technolog* OR technology adopt* OR technology integration OR e-learning OR elearning OR web-based OR web based OR Twitter OR tweet* OR Facebook OR podcast* OR blog*).mp.
AND
(health professional* OR physiotherap* OR physical therap* OR allied health).mp.

### Selection of the studies

**Fig. 1:**
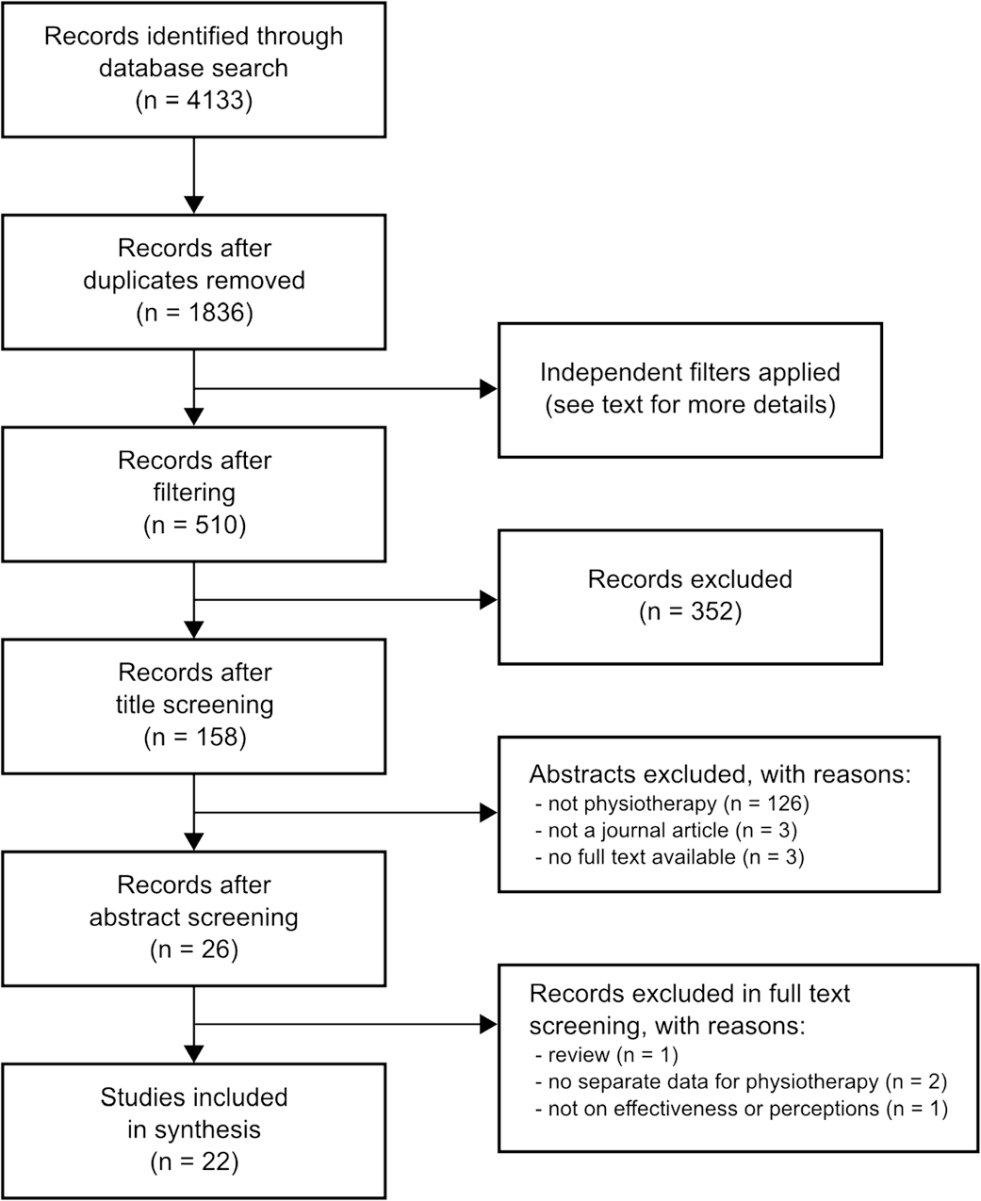
Search strategy and flow of the studies. (Prepared in accordance with PRISMA guidelines [17])

The process of study selection is presented in Fig. 1. After duplicates removal, title and abstract screening, were performed with use of the bibliographic software EndNote X7.1 [18]. Two reviewers screened the references independently, with each adopting a different strategy for screening. In the first screening strategy, using EndNoteX6, one reviewer (AKM) applied two filters on all the records, namely ‘teaching’ and ‘learning’ , and then title- and abstract-screened. The second reviewer (DCR), also using EndNoteX6, applied the following filters: teaching, learning, higher education, physiotherapy or physical therapy. Records for each filter were combined, and title- and abstract-screened for eligibility. Studies identified as eligible, were then compared with those identified by the first reviewer. Any disagreement regarding inclusion of articles was discussed to reach consensus. Remaining articles were then full-text screened and analysed.

### Assessment of risk of bias

Risk of bias was assessed using the 2011 Mixed Methods Appraisal Tool checklist (MMAT) [19], which allows for the assessment of studies with quantitative, qualitative, and mixed methodological designs. This checklist consists of two initial questions applicable to all study designs, and four questions applicable to each of the designs. Only the questions relevant to each study design are scored, and the number of ‘yes’ answers to these questions summed. This number is divided by the total number of relevant questions and multiplied by 100, giving a final percentage score.

Risk of bias in individual studies was independently assessed by two reviewers (AKM, DCR) and all discrepancies resolved by discussion to reach consensus. Inter-rater agreement on the risk of bias assessment was calculated by means of Cohen’s Kappa [20] using SPSS software v.16 [21].

### Data extraction and analysis

Studies were classified based on each of the two research questions of this review. One focused on the effectiveness of online technology to enhance learning and teaching in physiotherapy, and the other on the perceptions of learners on the use of technology. Study characteristics such as design, sample size and type, technology implemented, physiotherapy course, type of outcome measures and main findings, were extracted to a (pre-agreed) tabular form by one review author (AKM) and checked by the second review author (DCR). Disagreements were resolved by discussion between the two reviewers. Risk of bias was then assessed and added to the tables. The main findings from both groups of studies were analysed and narratively summarized.

Findings of the studies, designs and risk of bias were taken into account to draw the recommendations for some of the technologies.

## Results

### Search results

The flow and number of the studies screened through each stage of this review are presented in Fig. 1. A total of 4133 articles were retrieved. After excluding duplicates, 1836 articles were left, and underwent screening to leave 22 articles that met the inclusion criteria and were accepted for final analysis.

### Study characteristics

**Fig. 2:**
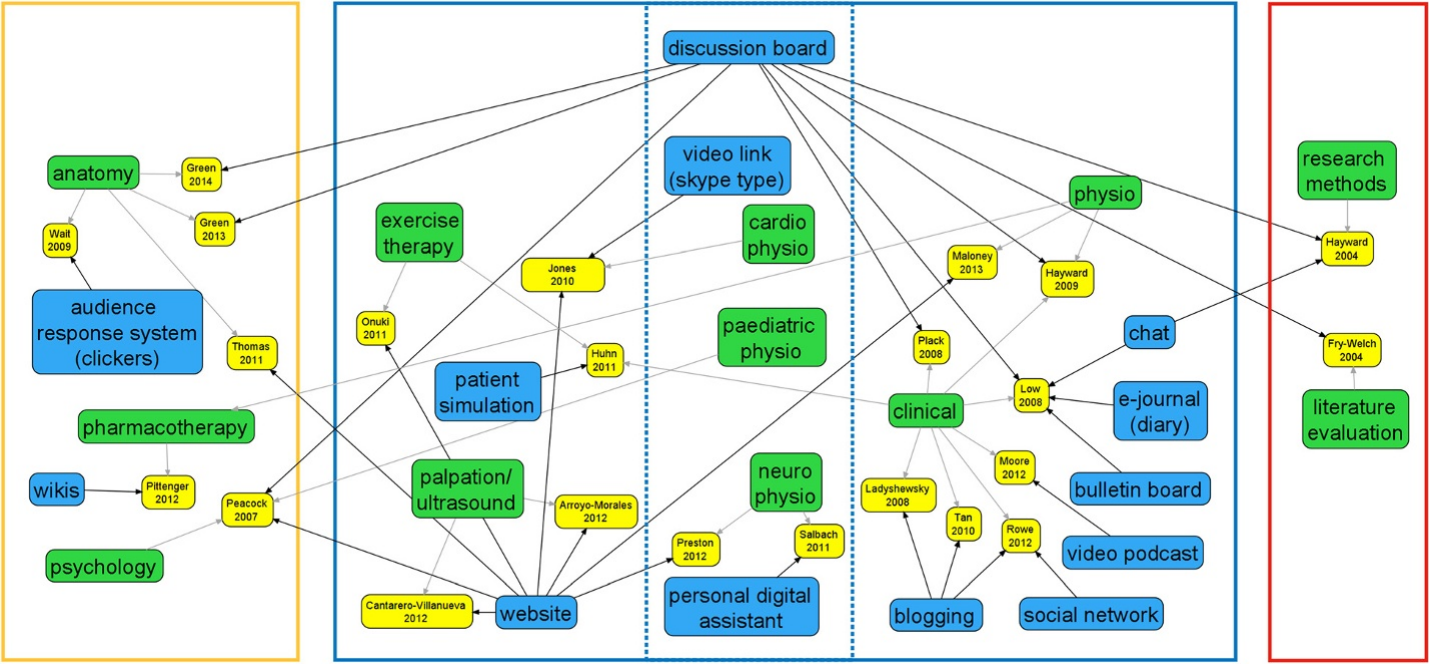
Map of technologies use in physiotherapy teaching and learning. (Green – disciplines in physiotherapy, blue – technologies used, yellow – studies; physio – physiotherapy)

All articles were published in English, but original studies were conducted in seven countries (i.e., Australia, Canada, Hong Kong, South Africa, Spain, the UK, and the USA). In general, studies used three designs: case study (14 articles), controlled trial (3), and randomized controlled trial (5). In total, included studies investigated 1694 participants, both pre-registration physiotherapy students (baccalaureate (1182), doctor of physical therapy (341), postgraduate (148)) and physiotherapy professionals (23). Studies investigated the use of online technologies, principally including websites and discussion boards across basic sciences (e.g., anatomy), physiotherapy disciplines (e.g., paediatric physiotherapy), to physiotherapy research (e.g., research methods). Eight studies investigated the effectiveness of technology in teaching and learning in physiotherapy, seven studies explored perceptions of physiotherapy students or professionals related to the use of such technology, and a further seven studies investigated both aspects. The map of technologies used in physiotherapy teaching and learning is presented in Fig. 2.

### Quality of the articles

The quality of studies ranged from 67 to 100 % on the MMAT checklist, which can be considered moderate to excellent. More than half of the studies (68 %) received scores greater than 80 %. Cohen’s Kappa for inter-rater agreement was 0.75, indicating “substantial agreement” between raters (range 0.61–0.80) [22]. None of the studies were excluded from the analysis on the basis of the MMAT score.

### Results of the individual studies

Review results are presented in two domains related to the research questions: the effectiveness of online technology, and perceptions of online technology.

#### Effectiveness of technology on physiotherapy teaching and learning

A summary of individual studies investigating the effectiveness of technologies in physiotherapy teaching and learning is presented in Table 2. In total, 15 articles investigated effectiveness of technology in physiotherapy teaching and learning. The designs used included randomised controlled trial (RCT) (5 studies), non-randomised controlled trial (CT) (3 studies) and case study (CS) (7 studies). The most commonly investigated technologies were websites (5 articles) and discussion boards (7 articles). The remaining three articles described the use of video podcasts, collaborative wikis, and blogging, respectively.

**Table 2:** Main aspects of the studies (*n* = 15) investigating effectiveness of technology in physiotherapy learning and teaching

Study credentials	Country	Design	Participants	Intervention: Technology used, Theme/course, Length of the intervention	Outcome measures (tool/method)	Key findings	Quality
Arroyo-Morales, 2012 [23]	Spain	RCT: Exp: ECOFISIO website, Con: books & texts	46 (28f) UG, 2nd	Website; ‘Fundamentals of Physiotherapy’; 3 weeks	Theoretical knowledge acquisition (multiple-choice questionnaire), skills acquisition: palpation and ultrasound imaging of the knee (structured objective clinical evaluation (SOCE)),	No difference between groups in the acquisition of theoretical knowledge; Global SOCE scores higher for exp group; Exp group needed less time to palpate, took longer to acquire ultrasound image, but got higher scores for correctly positioning and managing the probe.	83
Cantarero-Villanueva, 2012 [24]	Spain	RCT: Exp: ECOFISIO website, Con: books & texts.	50 (29f) UG	Website; ‘Fundamentals of Physiotherapy’: Palpation and ultrasound; 2 modules	Skill of palpation and ultrasound imaging of lumbopelvic area (objectively structured clinical examination (OSCE));	Global scores for palpation and global scores of ultrasound examination significantly higher in the exp group; Exp group needed less time to obtain an ultrasound image.	83
Fry-Welch, 2004 [31]	USA	CS	64 (42f) DPT	Threaded discussion; ‘Critical Evaluation of the Professional Literature’; 4 weeks	Engagement (6 questions, 5-point Likert scale + additional comments)	Threaded discussion was effective in getting students to read articles before the class, increasing active participation in reflective discussion of journal articles before the class, letting professor to target in-class discussion on difficult issues; Not effective in engaging reflection on other students’ comments.	100
Green, 2014 [28]	Australia	CS, TG	97 (campus1) +41 (campus 2) UG, 2nd	Discussion forum; Gross a natomy for physiotherapy students; 1 semester	Contribution of participation (number of posts), previous academic ability (prerequisit grade), and campus to final grade	The total number of posts made a significant positive direct contribution to final grade.	100
Green, 2013 [29]	Australia	CS, TG	460 UG, 1^st^ 137 UG, 2^nd^	Discussion forum; Anatomy; 1 semester	Contribution of a number of posts to the final grade	In 1^st^ year students, there was no relation between number of posts and final grade. 2^nd^ year students who highly contributed (high number of posts) to the discussion forum obtained higher final grades.	100
Hayward, 2009 [34]	USA	CS (pre-post measurement)	104 (83f) DPT, 3rd	Virtual learning environment: case scenarios, discussion board (+ other); ' Physical Therapy Professional Seminar I'; 15-weeks course	Professional skill awareness (pre-post *Professionalism in Physical Therapy: Core Values* survey)	Significant improvement in students’ awareness of professional skills.	92
Huhn, 2011 [25]	USA	RCT: Exp: web-based cases, Con: text-based cases	36 UG	Web-based simulation cases; 'Therapeutic Exercise'; 3 stimulation cases	Clinical reasoning skills (Health science reasoning test (HSRT)), knowledge transfer (practical exam), time spent on case completion, implementation costs	No significant difference between the groups in the total HSRT; Exp group scored better on practical exam, and spent less time per case. Cost of computerized simulation was lower in implementation.	67
Jones, 2010 [26]	Canada, Hong Kong	TG, RCT: Exp1: video-linked tutorials + web-based tutorials, Exp2: web-based tutorials, Con: lecture tutorial	Canada: 35, Hong, Kong:37	Video link, web-based tutorials; Two topics: oxygen transport, manual hyperinflation (cardiovascular and cardiopulmonary physiotherapy); 3 weeks	Knowledge (grades on objective short-answers quiz)	No differences in mean scores for the two topics across the 3 intervention formats with an exception of web-based Hong Kong group which scored lower in oxygen transport.	67
Low, 2008 [30]	USA	CT: Exp: internship with virtual learning environment, Con: internship without virtual learning environment	81 PG	Virtual learning environment: chat, threaded discussion, bulletin board, e-diary; 1 clinical internship	Critical thinking skills (California CT Skills Test (CCTST)	No differences in CCTST between groups.	100
Moore, 2012 [37]	USA	RCT: Exp: lecture + podcast demonstration, Con: lecture + live demonstration	33 DPT, 1st	Video podcasts; Basic clinical skills: transfer and gait training; 3 weeks	Cognitive performance (written test), psychomotor performance (scenario-based practical exam), study time (self-reported)	No significant differences in written and practical exam scores between methods. Group receiving podcasts reported more group study time than live demonstration.	67
Pittenger, 2012 [35]	USA	CS	50 DPT,2nd	Collaborative wikis; ‘Rehabilitation Pharmacotherapy’; 1 semester	Learning (grades distribution in comparison to previous years)	Grades distribution consistent with grades from previous years when technology was not used.	77
Plack, 2008 [33]	USA	CT Exp1: mentor-facilitated, Exp2: peer-facilitated	7 DPT, PG	D iscussion board; A cute rehabilitation internship; 1 clinical rotation	Reflective thinking and higher-order processing (number of entries to the discussion board, evidence in posts for reflective thinking and higher-order processing)	No differences between groups in reflective thinking, and conclusion drawing; Exp1 group exhibited greater proportions of data gathering, and data analysis levels of higher-order thinking; Exp2 group submitted higher number of advice responses.	100
Preston, 2012 [27]	Australia	CT Exp: online teaching additional to usual teaching, Con: usual teaching	59 UG,2nd	D igital repository; N eurological physiotherapy; 5-weeks + revision session	Performance of practical skills (standardised marking schema)	Exp group scored higher in the practical exam.	100
Rowe, 2012 [32]	South Africa	CS, TG	70 UG 3^rd^,4th	Discussion board, social network, blogging; Clinical placements; 1 academic year	Reflective reasoning (assignment related student-teacher, student-student, teacher-student interactions, thematic analysis)	Evidence found for online social networks to develop reflective practices among students.	67
Tan, 2010 [36]	Australia	CS	45 UG, 4^th^ (final)	Blogging; Clinical placement	Clinical reasoning and metacognition (content analysis, proof of event counts)	Proof of a range of clinical reasoning and metacognitive skills.	83

*RCT* randomised controlled trial, *CT* clinical trial, *CS* case study, *TG* two group design, *UG* undergraduate programme, *DPT* doctor of physical therapy programme, *PG* postgraduate programme, *PR* physiotherapy professionals, *f* female, *Exp* experimental group, *Con* control group, *2*^*nd*^ second year students, *3*^*rd*^ third year students, *4*^*th*^ fourth year students, *5*^*th*^ fifth year students

##### Websites

Effects of websites on practical skills performance was investigated in five studies: four RCTs [23–26, 27] and one CT [27]. The websites included web-based tutorials, or online repositories with videos and patient-therapist simulations. All showed an improvement in practical skills in the groups using websites. Findings from three RCTs [23–25] suggested also that groups using websites needed less time to perform a task; a single RCT [23] reported the website group to require more time for performing a task. Two RCTs [23, 26] found no differences between groups’ knowledge acquisition. One RCT [25] showed no differences between traditional text-based cases and website simulations for teaching clinical reasoning, but reported lower costs associated with the implementation of website (simulations) when compared to traditional teaching costs.

##### Discussion boards

Seven studies explored the effect of discussion boards on knowledge acquisition, as well as on development of critical and reflective thinking. Two case studies [28, 29] found significant improvements in knowledge acquisition (measured by students’ final marks) through the use of discussion boards. Two other studies investigated influence of discussion boards on critical thinking. Results from a controlled trial [30] suggested a significant improvement in critical thinking in the experimental group (exposed to a discussion board) compared to the control group (not exposed to a discussion board). A case study [31] similarly indicated enhanced critical thinking related to literature evaluation. Another case study [32] reported positive effects on reflective practices, and one controlled trial [33] found evidence of benefits in reflective thinking (with no difference between peer-facilitated and mentor-facilitated discussion boards). Peer-facilitated and mentor-facilitated discussion boards were both found to facilitate conclusion drawing, but a greater degree of higher-order thinking in mentor-facilitated group was observed [33]. Other case studies reported discussion forums being effective in increasing active participation in discussion of journal articles [31] and in increasing students’ awareness of core professional values [34].

##### Other technologies

Case studies have shown no improvement in grades using collaborative wikis [35], but that students demonstrated a range of clinical reasoning and metacognitive skills using blogging [36]. Another RCT study [37] using podcasts found no significant effect on written nor practical exam scores.

#### Perceptions of benefits and barriers for use of technology in physiotherapy teaching and learning

A summary of individual studies investigating perceptions related to online technology use in physiotherapy teaching and learning is presented in Table 3. Perceptions of physiotherapy students and professionals on technologies used were investigated in 14 articles, of which four were randomised controlled trials, two were non-randomised controlled trials, and eight were case studies.

**Table 3:** Main aspects of the studies (*n* = 14) investigating perceptions of students/users on technology in physiotherapy learning and teaching

Study credentials	Country	Design	Participants	Intervention technology used, Theme/course, Length of the intervention	Outcome measures (tool/method)	Key findings	Quality
Arroyo-Morales, 2012 [23]	Spain	RCT	46 (28f) UG, 2nd	Website; ‘Fundamentals of Physiotherapy’; 3 weeks	Perceptions on the quality of the educational environment (1–5 Likert)	Exp group scored higher on 3/10 items: ‘classes were entertaining’ , ‘I was able to learn a lot’ , ‘I was able to apply what I learned’; Con group scored higher on willingness to learn another anatomical region. Exp group reported high levels of satisfaction with the website.	83
Cantarero-Villanueva, 2012 [24]	Spain	RCT	50 (29f) UG	Website; ' Fundamentals of Physiotherapy': palpation and ultrasound; 2 modules	Students perceptions of quality of educational method (5-point Likert survey)	No differences between groups in participants’ evaluation of the quality of learning.	83
Hayward, 2004 [41]	USA	CS	57 PG,5th	Virtual learning environment: chat, discussion board; ‘Research for Physical Therapists’; 12-week course	Experiences and perceived learning (discussion board and chat room transcripts, reflective papers)	Discussion boards were deepening thinking and ability to critically examine a topic, also gave participants multiple perspectives. Technology was perceived as beneficial for improving self-directed learning strategies. Barriers included problems with access to the internet or computer, and preference for face-to-face contact.	100
Jones, 2010 [26]	Canada, Hong Kong	RCT, TG	Canada: 35, Hong, Kong:37	Video link, web-based tutorials; Two topics: oxygen transport, manual hyperinflation (cardiovascular and cardiopulmonary physiotherapy); 3 weeks	Students evaluation of learning experience	Video link group valued learning from international peers.	67
Ladyshew-sky, 2008 [42]	Australia	CS	32 UG, 4th	Blogging; Clinical placements; 15 weeks	Perceptions of learning experience and support for reflective practice	Students liked simplicity, non-threatening environment, informality, accessibility and convenience of blogging; they enjoyed learning from each other. Blogging required reflection, processing of thoughts and structuring them; let for building the trust in the group. Perceived barriers included: technical issues, too small groups, lack of example blogs.	67
Low, 2008 [30]	USA	CT	81 PG	Virtual learning environment (VLE): chat, threaded discussion, bulletin board, e-diary; clinical internship; 6 or 12 weeks	Communication with classmates and perceived effectiveness of program (survey)	VLE: let for communication, was easy to use; e-diary: beneficial source of reflection. Technology was supportive for students while off campus.	100
Maloney, 2013 [38]	Australia	CS	18 UG, 4^th^	Digital repository; placements; semester	Attitudes (focus groups)	Online resources convenient and useable; could support physiotherapy practice in workplace; may be an effective tool for life-long learning.	85
Moore, 2012 [37]	USA	RCT	33 DPT, 1st	Video podcasts; Basic clinical skills course: transfer and 3 weeks	Perceptions of use of both learning methods (survey with 1–5 Likert scales)	Podcasting appeared to be a reasonable alternative to in-class demonstrations for teaching basic transfer and gait training skills.	67
Peacock, 2007 [39]	UK	CS, TG	49 UG, 10 PG	Digital repository, discussion board; psychology (UG), paediatric (PG);1 semester	Perceptions, expectations, views (students, tutors focus groups and interviews)	Discussions provided peer-support and encouraged engagement with learning materials. Issues such as access, induction, IT skills need, and time requirement, have to be addressed before e-learning will offer ‘another dimension’ to lifelong learning.	100
Pittenger, 2012 [35]	USA	CS	50 DPT,2nd	Collaborative wikis; 'Rehabilitation Pharmacotherapy'; 1 semester	Previous experience with online learning (survey),	Wikis were successful in learning pharmacotherapy. Writing was helpful and difficult, complexity of the course form allowed for pharmacotherapy application in physiotherapy context.	77
					perceived effectiveness and feasibility of the course form (survey, focus groups);		
					professional identity development (reflection assignment, focus groups)		
Preston, 2012 [27]	Australia	CT Exp: online teaching additional to usual teaching, Con: usual teaching;	59 UG,2nd	Digital repository; Neurological physiotherapy; 5-weeks + revision session	Perceived usefulness of the resource (survey with VAS + comments)	Resource useful, handy, great visual tool and reminder of what was learnt in class.	100
Salbach, 2011 [43]	Canada	CS	23 PR	Personal digital assistant; Neurophysiotherapy; one off	Preferences for strategies to increase access to, implementation and application of research findings into clinical practice (in depth telephone interviews)	Advantages of PDA: quick and timely access to information, integration of information, sharing. Concerns: PDA may not be available in workplace, requirement to wear it, patient perception on technology use during a visit, high cost, training how to use it required.	83
Thomas, 2011 [40]	USA	CS	25 + 30 DPT,2st	Website; ‘Human gross anatomy’; 10 weeks	Perceptions (pre-, post-questionnaire Likert scale 1–5)	Students benefited from multimodal approach to learning.	83
Wait, 2009 [44]	USA	CS	28 DPT,1st	Audience response system (ARS); ‘Anatomy for Physical Therapists’; 16 weeks	Perceptions (Likert 1–5 + open ended questions)	ARS permitted for self-assessment and comparison of the performance with others; the immediacy of ARS feedback enhanced students' confidence to actively participate in subsequent small group discussions.	77

*RCT* randomised controlled trial, *CT* clinical trial, *CS* case study, *TG* two group design, *UG* undergraduate programme, *DPT* doctor of physical therapy programme, *PG* postgraduate programme, *PR* physiotherapy professionals, *f* female, *2*^*nd*^ second year students, *3*^*rd*^ third year students, *4*^*th*^ fourth year students, *5*^*th*^ fifth year students, *VLE* virtual learning environment, *PDA* personal digital assistant, *ARS* audio response system

##### Websites

Seven studies investigated users’ perceptions of websites focusing mostly on positive feedback [23, 24, 26, 27, 38–40]. Websites contained repositories of course related materials, web-based tutorials, and also videos and quizzes. Participants’ feedback described the use of websites as entertaining, easy to access, ‘great for learning’ [27], and a beneficial tool. Websites allowed participants to access all materials in one place, and enabled enjoyable, independent learning. One of participants stated: ‘I found that everything was there that you needed… you didn’t need to go and ask’ [39]. Only two studies explored barriers in using websites [26, 39]: common issues identified included difficulties with internet connection, insufficiently interactive material, or simply preference for paper-based materials.

##### Discussion boards

Three studies investigated feedback related to the use of discussion boards [30, 39, 41]; all combined analysis of discussion boards with other technologies such as chat, website, or e-diary. Discussion boards were perceived as deepening the thinking and facilitating reflection, allowing for learning from multiple perspectives, and facilitating communication and support. Also, discussion boards allowed students to ‘collect their thoughts before responding’ while ‘not put on the spot’ [41].

##### Other technologies

Users’ perceptions of other online technologies were investigated in eight studies, which investigated chat [41], video link [30], blogging [42], e-diary [30], bulletin [30], video podcasts [37], collaborative wikis [35], personal digital assistants [43], and an audience response system [44]. In general, these technologies facilitated communication, reflection, quick and timely access to information, self-assessment and comparison to others.

## Discussion

This study is the first to systematically review the effectiveness of, and users’ perceptions of, online technology in physiotherapy teaching and learning. This review included a total of 22 articles (15 on the effectiveness of technology, and 14 on users’ perceptions of the use of technology). Articles included in this review used different methods (e.g., quantitative, qualitative, and mixed methods), study designs (RCT, CT, case study), and outcome measures. Therefore, it was not possible to conduct meta-analysis. Despite the heterogeneity of study designs, interventions, and outcome measures, all included studies were considered as of low risk of bias.

The effectiveness of interventions varied according to the technology used (Table 2). Websites improved practical skills performance; however, their effect on time to perform a given task is inconclusive. Furthermore, no added benefit was found with the use of websites on knowledge acquisition or clinical reasoning; however, their use resulted in lower costs. In contrast, discussion boards were found to improve knowledge acquisition, critical and reflective thinking, and increase students’ awareness of core professional values and active participation in discussion on journal articles. Overall, our results suggest benefits of websites and discussion boards for facilitating learning, and enhancing the development of practical skills and knowledge acquisition (although it is noted that two studies reported no difference between knowledge acquisition using websites).

Users’ perceptions of the use of technologies for teaching and learning purposes were mostly positive (Table 3). Users acknowledged technologies such as websites and discussion boards for allowing access to all relevant materials in one place, for representing an enjoyable and entertaining learning experience, for facilitation of thinking, reflection, and learning from multiple perspectives. However, this positive view may be biased due to paucity of studies focusing on barriers to online technology use.

Results from this review are in agreement with previous reviews investigating the use of online and computer-related technologies in medical, dental, and nursing education. Previous reviews have shown equal or superior learning outcomes with the use of online technologies [11, 15] with few studies showing negative results for the use of web-technologies in teaching and learning [12, 13]. Similarly to this review, previous reviews conducted with other health professions have shown that most studies reported positive attitudes from medical, dental and nursing students towards technology [15]. Combined, findings from the present and previous reviews suggest that technology has a place in health professional education, but its incorporation into learning and teaching practices needs to be carefully planned.

From the date the searches were commenced for the current review, five additional articles have been published on the topic. These five articles were case studies, four were published in English language and one article was published in French [45]. Two studies investigated the use of videos as a way of gathering feedback on practical skills performance [46] or delivering lectures [47]. The outcomes suggested positive students’ perceptions towards these teaching approaches. Two other studies investigated blogging for the development of reflection on practice. Blogging elicited reflection on students’ practice; however the results suggested some barriers, such as technical difficulties in posting [48]. Blogging also facilitated the development of reflection and research skills [49]. The fifth study [45] investigated the use of simulation software on development of clinical reasoning in cardiorespiratory physiotherapy. The reported results suggested students perceived it as a positive and satisfactory method. These additional five studies reported similar findings to those identified through our original search, suggesting that web-technologies may be useful instruments for strengthening teaching methods in undergraduate and postgraduate levels.

Research on the use of web-based technologies in physiotherapy is at its infancy, which impacted on the present review in several respects. First, the included studies had different study designs, outcome measures, used different technologies (interventions), and explored the use of web-based technologies in different physiotherapy courses and populations. Therefore, it is difficult to identify the optimum approaches and practices for the use of web-based technology on teaching and learning in physiotherapy. It also precluded meta-analysis of results. Second, only five out of 15 studies were RCTs, limiting the classification of the level of evidence regarding the effectiveness of interventions being evaluated. Finally, the definition of online technologies used in this study might not have been exhaustive and this is recognised as limitation. This field of research is rapidly evolving with a paucity of studies and varying definitions. There is a need for clarification of the definitions as the literature expands.

Future research should focus on the identification of barriers for the use of online technologies for teaching and learning in physiotherapy. Addressing such barriers may optimize adherence to the use of online technologies, enhancing learning outcomes. Also, physiotherapy education would benefit from studies evaluating individual online technologies in robust methodologically designs (e.g., added benefit type RCT), and the adoption of reporting guidelines such as CONSORT [50] or COREQ [51].

## Conclusions

The results of this review suggest that online technologies (i.e., websites and discussion boards) have many benefits to offer for physiotherapy teaching and learning. These include enhancing practical skills performance and knowledge acquisition, providing students with entertaining, easy accessible resources, enhancing deep learning and encouraging reflection. Such benefits allow for learning to occur from multiple perspectives. Perceived barriers included difficulties with internet connection, insufficiently interactive material, or personal preference for paper-based materials. Although there was minimal evidence of barriers for the use of online technologies, addressing the identified ones could enhance adherence to use of online technologies in health professionals’ education.
